# A longitudinal controlled signage intervention to increase stair use at university buildings: Process and impact evaluation using RE-AIM framework

**DOI:** 10.3389/fpubh.2023.1079241

**Published:** 2023-04-18

**Authors:** Zaenal Muttaqien, Widya Wasityastuti, Meida Sofyana, Denny Agustiningsih, Rakhmat Ari Wibowo

**Affiliations:** ^1^Department of Physiology, Faculty of Medicine, Public Health, and Nursing, Universitas Gadjah Mada, Yogyakarta, Indonesia; ^2^Physical Activity for Health Research Centre (PAHRC), Institute for Sport, Physical Education and Health Sciences, Moray House School of Education and Sport, University of Edinburgh, Edinburgh, United Kingdom

**Keywords:** community-based participatory research, health promotion, motor activity, stair climbing, video recording, workplace

## Abstract

**Introduction:**

Stair climbing intervention could be suggested to address low occupational physical activity amongst university students and employees. Strong evidence showed the effectiveness of signage intervention in increasing stair use in public areas. However, evidence in worksite settings, including university settings, was inconclusive. This study aimed to evaluate the process and impact of a signage intervention to increase stair use at a university building using the RE-AIM framework.

**Method:**

We conducted a non-randomised controlled pretest-posttest study to examine the effect of signage intervention placed in university buildings in Yogyakarta (Indonesia) between September 2019 and March 2020. The process of designing the signage involved the employees in the intervention building. The main outcome was the change in the proportion of stair use to elevator use measured by manual observations of video recordings from closed-circuit television. A linear mixed model examined the intervention effect by controlling the total visitor count as a confounder. RE-AIM framework was used in the process and impact evaluation.

**Results:**

The change in the proportion of stair climbing from baseline to the 6th-month phase at the intervention building (+0.067 (95% CI = 0.014–0.120)) was significantly higher than that of the control building. However, the signs did not change the proportion of the stair descending at the intervention building. The signs were potentially viewed 15,077–18,868 times/week by visitors.

**Conclusion:**

Signage intervention using portable posters could easily be adopted, implemented, and maintained in similar settings. A co-produced low-cost signage intervention was found to have a good reach, effectiveness, adoption, implementation, and maintenance dimension.

## 1. Introduction

Several studies showed that office workers and university students were at health risk due to the high occupational sitting time and low occupational physical activity (PA) (1–4). Stair climbing, which was categorised as vigorous PA, has been associated with health benefits amongst both healthy individuals and also individuals with health conditions, including improvements in aerobic capacity, blood pressure, lipid profiles, body composition, mood states, cognitive performance, and reduced risk of several non-communicable diseases and mortality (5–13). Thus, an intervention that motivates stair climbing in university settings could be suggested to increase occupational PA to improve office workers’ and students’ health.

Several systematic reviews reported that motivational signage posted near stairs and elevators could increase stair use in public areas (14–16). However, the effectiveness of stair use intervention in worksite buildings was not consistent, particularly in university-based settings (14, 17–20). In addition, contemporary phenomena could interfere with the effect since there was no control group in previous university building studies (19, 20). Most of the available studies on the effectiveness of signage also had weak internal validity and lacked external validity reporting (14–20).

Our study aimed to examine the effectiveness of motivational signage using a specific message on stair use and to report its process evaluation using the RE-AIM (Reach, Effectiveness, Adoption, Implementation, and Maintenance) Framework to improve its external validity and to inform future trials (21). We examined the outcome in an identical building as a control to minimise the bias from contemporaneous phenomenons. Objective measurement using video recording by two independent observers blinded to the intervention status was employed to reduce measurement bias and Hawthorne effects.

## 2. Methods

**Figure 1:**
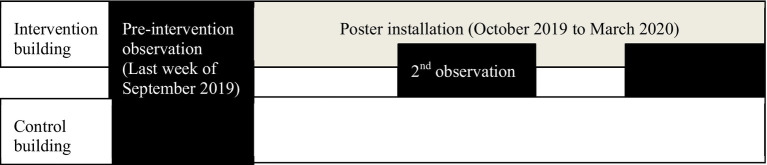
Study flow.

A longitudinal non-randomised controlled quasi-experimental study was conducted to assess the change in stair use for stair climbing or descending as an effect of point-of-decision motivational signage (Figure 1). The study was retrospectively registered at thaiclinicaltrials.org (TCTR20220804005) and was ethically approved by the Medical and Health Research Ethics Committee Faculty of Medicine, Public Health, and Nursing Universitas Gadjah Mada (Ref No: KE/FK/0973/EC). This pragmatic trial was reported in line with the Consolidated Standards of Reporting Trials (CONSORT) statements for pragmatic, pilot, and feasibility trials (22, 23). This study consisted of 1-week pre-intervention data collection to measure the baseline stair use and elevator use, followed by 6 months period of intervention monitoring (October 2019–March 2020). The second monitoring phase was conducted in the last week of the 3rd month, and the third phase was conducted in the last week of the 6th month.

### 2.1. Sample

This study was conducted in two university buildings in Universitas Gadjah Mada, Yogyakarta, Indonesia. They were located 260 m apart in two different faculties in the health science cluster. Employees and students rarely moved from one building to another, which could minimise the risk of contamination bias. In both faculties, a health promotion initiative called the Health-Promoting University (HPU) has been running since July 2019. The same team supervised health promotion programs at the two sites. They targeted the same fields of intervention, including health literacy, physical activity, nutrition, mental health, zero tolerance for smoking, alcohol consumption, and illicit drug use, zero tolerance for violence, bullying, and sexual harassment, and safe buildings and healthy environments.

In choosing the intervention and control buildings, the following characteristics were sought: (1) Two buildings with an almost similar number of occupants, (2) Two buildings with almost similar staircase and elevator design and placement, as well as the numbers of the stories (Table 1). We did not randomly assign the status of the intervention and control building because there was already an A6 motivational sticker installed just above the elevator for 3 months.

**Table 1:** Characteristics of the buildings and posters.

	Control site	Intervention site
Number of employees	65	90
Type of employees’ activities	Laboratory employees	30 of them are administration employees, and the rest are laboratory employees
Number of students	894	902
Type of students’ activities	Laboratory works	Laboratory works
Number of floors	6	5
Stair and elevator location	Centrally located circular stair with a central elevator in a large atrium and an elevator at the opposite side	Centrally located circular stair with a central elevator in a large atrium and an elevator at the opposite side
Stair landing	2	3
Lift capacity	13 persons	11 persons
Number of lift	2	2
Poster location	–	Placed both next to elevators and also to stairs
Poster size		1.6 × 0.9 m

### 2.2. Measures

Manual observations from video recordings of closed-circuit television (CCTV) were independently conducted from Monday to Friday in each phase by two observers blinded to the intervention status. Therefore, visitors were not aware of being observed. The speed of the video recordings was accelerated two times to reduce observers’ burden, and the resolution was lowered to protect visitors’ privacy. In addition to counting the visitor journey, whether entering the lift, exiting the lift, climbing the stairs, or descending the stairs, observers also took note of the incidence of stair accidents. The two observers statistically significantly agreed in their counts (*W* = 0.998, *p* < 0.001). The daily observations in workdays per 2-h time slot (07.00–09.00, 09.00–11.00, 11.00–13.00, 13.00–15.00, 15.00–17.00) were written down in a spreadsheet at all locations. The proportion of stair use per time slot was calculated by dividing the number of people taking the stair from the ground floor by the number of people entering the elevator as a proportion of stair climbing and by dividing the number of people taking the stair to the ground floor by the number of people leaving the elevator as a proportion of stair descending.

### 2.3. Analysis

The total ascending journey from the ground floor and descending journey to the ground floor were reported and grouped by the use of a staircase or elevator. Mann–Whitney *U*-tests were conducted to examine the baseline proportion of stair climbing and stair descending per time slot between intervention and control building. The intervention effects as a mean change per time slot in the intervention building compared to the control building were examined using linear mixed models (24). Building (intervention, control) and period (1–3, corresponding to the baseline period, 3rd-month phase, and 6th-month phase) and total visitors per 2-h time slot as covariates were included in the models as fixed effects. Interaction terms between building and period variables were added (effects at the intervention site compared to the control site for all three study periods). Results are reported as differences in mean absolute change with the 95% confidence interval (CI). Analyses were performed with SPSS version 25.

### 2.4. Process and impact evaluation

We performed process evaluation using RE-AIM (Reach, Effectiveness, Adoption, Implementation, and Maintenance) Framework (21). Reach was the total number of visitors counted over time during the study period. Effectiveness was measured by the effects of the intervention on the change of stair use proportion from baseline in the intervention building compared to the change in the control building. Adoption was described as the participation of employees in the intervention building in defining and implementing the signage and barriers to participation. The implementation dimension was examined based on the extent to which the intervention was delivered as intended. The maintenance dimension was how stair-use interventions were sustained at the intervention building over time. The head of the HPU unit and the dean are two stakeholders in the intervention building who will adopt, implement and maintain the intervention. Therefore, the adoption, implementation, and maintenance dimension were evaluated based on an informal interview with the employees using guided questions adopted from a previous study (25). The interview consisted of questions asking how the stakeholders describe the implementation of the lift signages placement, the main barriers during the placement, any difference in intervention delivery from those suggested by the researchers team, as well as their plan at the end of the study period. Informal opinions from the HPU unit from the other faculties were also captured during the dissemination of the intervention at the end of the study period through an internal social media group.

### 2.5. Intervention and the co-production stage

**Figure 2:**
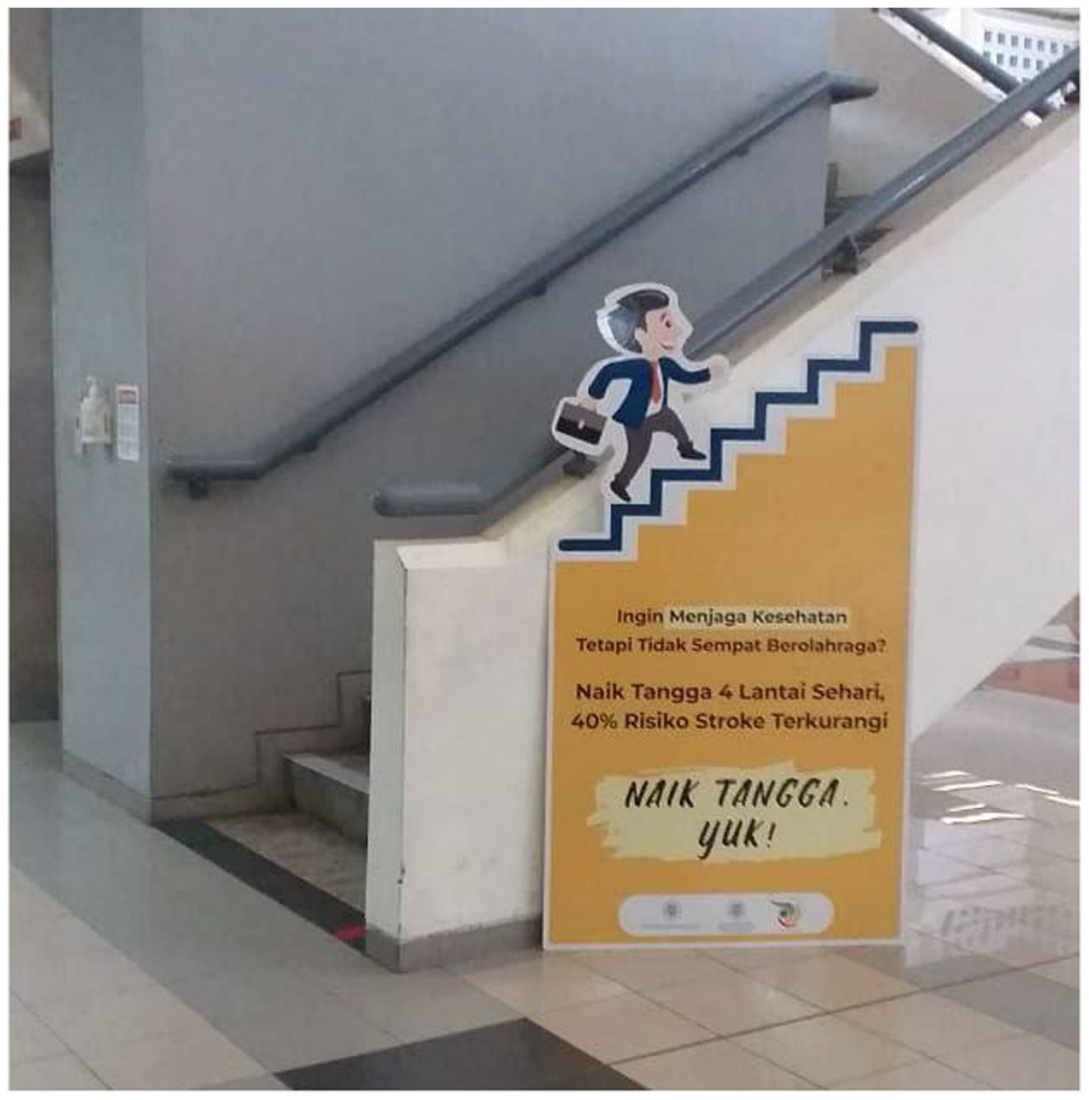
Placement and design of the poster.

We used a co-production approach by involving employees at the end of the value chain to get the optimal benefits of a collaborative approach whilst minimising resource constraints (26). A survey was sent to the employees in the intervention building to pre-test the motivational messages. Three specific messages were pre-developed since they provided better effectiveness than general messages (20). Open-ended questions were asked for their opinions and feedback on the messages and designs. Six of ninety employees provided answers and returned the letter. Thematic analysis from employees’ opinions and feedback was described (Table 2). The health-related message was chosen since no negative theme arose from this message. Therefore, two portable posters of 1.6 × 0.9 m containing a health-related message and a picture representing an employee climbing the stairs were placed next to the staircase and the elevator on the ground floor of the intervention building (Figure 2).

**Table 2:** Employees’ opinions on motivational texts.

Message type	Motivational text	Positive theme	Negative theme
Health-related message	Do you want to maintain health but do not have time to exercise? Climb 4 floors a day, and you get a 40% reduction in stroke risk Take the stairs! (In Bahasa Indonesia: Pingin Jaga Kesehatan, Tapi Nggak Sempat Olahraga? Naik Tangga 4 Lantai Sehari, 40% Risiko Stroke Terkurangi Naik Tangga Yuk!)	Motivating, Enhancing health awareness	
Weight-related message	Do you want to maintain weight but do not have time to exercise? Every time you climb 1 Floor, you burn 2.5 Calories. Take the stairs! (In Bahasa Indonesia: Pingin Jaga Berat Badan, Tapi Nggak Sempat Olahraga? Naik Tangga 1 Lantai, Bakar 2.5 Kalori Naik Tangga Yuk!)	Motivating, Easy physical activity	Unimportant
Time pressure	Are you waiting in line for the elevator and afraid to be late? It only takes 15 s to go up 1 floor by stairs. Take the stairs! (In Bahasa Indonesia: Antri Naik Lift, Tapi Keburu Terlambat? Hanya Butuh 15 Detik Untuk Naik 1 Lantai Dengan Tangga Naik Tangga Yuk!)	Motivating	Unimportant, Fear of sweaty

## 3. Results

### 3.1. Reach and effectiveness of intervention

A total of 93,000 counts from 75 observations resulting from five sessions per day for 5 days during three periods were analysed. There were 28,823 staircases and 64,177 elevator uses on the ground floor of two observed buildings, and no stair accidents occurred. The total visitor counts at the 3rd-month phase were lower than the baseline and 6th-month phases at both buildings (Table 3). In the intervention building, the stair posters have the potential to be viewed 15,077 times/week in the 3rd-month phase and 18,868 times/week in the 6th-month phase.

**Table 3:** Stair and elevator use.

		Baseline		3rd month		6th month	
		Ascending	Descending	Ascending	Descending	Ascending	Descending
Control Building	Stair	1,644	2,565	631	1,138	1,655	2,524
	Elevator	5,963	4,674	4,764	4,182	5,940	4,728
	Total	7,607	7,239	5,395	5,320	7,595	7,252
	Stair use proportion	0.216	0.549	0.117	0.214	0.218	0.348
Total journey		14,846		10,815		14,847	
Intervention Building	Stair	2,984	4,035	1811	2,549	3,178	4,110
	Elevator	6,412	5,310	5,753	4,964	6,252	5,238
	Total	9,396	9,345	7,564	7,513	9,430	9,348
	Stair use proportion	0.318	0.432	0.239	0.339	0.337	0.440
Total journey		18,741		15,077		18,868	

The stair climbing rate per time slot from the ground floor in the intervention building during the baseline phase was higher than that in the control building (*p* < 0.001), with an unadjusted median of 0.408 (0.12–1.03) and 0.224 (0.03–0.52), respectively. In the 3rd month phase, there were decreases in the stair climbing rate per time slot in both buildings, but there was no significant difference in the change between the two buildings. In the 6th month phase, the stair climbing rate per time slot in the intervention building significantly increased by +0.067 but no change in the control building (Table 4). Total visitors during the 2-h time slot had an estimated effect of 0.0003 (95% CI 0.0001–0.0004, *p* < 0.001) on the proportion of stair climbing per time slot.

**Table 4:** Change in the proportion of stair use to elevator use from the ground floor (stair climbing rate) per time slot.

	Intervention	Control	Intervention effect	*p* *	*p* **
Baseline					
3rd month	−0.170 (−0.224 to −0.117)	−0.103 (−0.162 to −0.043)	−0.001 (−0.063 to 0.061)	0.975	
6th month	0.067 (0.014 to 0.120)	0.002 (−0.047 to 0.050)	0.065 (0.005 to 0.125)	0.033	0.049

* Effect of building.

** Effect of period.

There was a significant difference (*p* < 0.001) between the stair descending rate to the ground floor per time slot in the intervention building (median = 0.721, 0.32–1.24) and the control building (median = 0.474, 0.12–1.50) during the baseline phase. There were decreases in descending stair proportion per time slot at the 3rd-month phase and returned to the baseline at the 6th-month phase in both intervention and control buildings. There was no significant difference in the changes between those two buildings (Table 5). Total visitors during the 2-h time did not have a significant estimated effect on the proportion of stair descending per time slot (*p* = 0.312).

**Table 5:** Change in the proportion of stair use to elevator use on the ground floor (stair descending rate) per time slot.

	Intervention	Control	Intervention effect	*p* *	*p* **
Baseline					
3rd month	−0.221 (−0.315 to −0.127)	−0.262 (−0.365 to −0.158)	0.041 (−0.068 to 0.149)	0.461	
6th month	0.072 (−0.022 to 0.166)	−0.030 (−0.115 to 0.056)	0.102 (−0.004 to 0.207)	0.059	0.164

* Effect of building.

** Effect of period.

### 3.2. Adoption

Two faculty employees participated in the design and implementation of the stair-use intervention. The implementation of the intervention was described as easy. They found that the stair posters could be easily installed at the point of choice and moved to another place if other posters need to be placed in the same location since the motivational prompts were portable standing posters.

### 3.3. Implementation

Employees could easily implement the recommended strategy from the research team to introduce the posters at the point of choice without changing the strategy. The intervention cost was 450,000 IDR for each poster and was paid by the research team’s funding. Employees in the intervention building considered the intervention’s cost affordable.

### 3.4. Maintenance

The two stair posters were maintained after the end of the study. Considering the easy-deliver and low cost of the intervention, the employees in the intervention building planned two additional intervention phases to test the effect of different new posters with different messages designed by involving students’ opinions and improving the stairwell’s aesthetic by installing colourful stair stickers. Informal opinions from the HPU units from the other faculties suggested that the intervention will be implemented in their faculties.

## 4. Discussion

We described the process of designing the stair-use intervention and evaluating the intervention using the RE-AIM framework (21). A low-cost motivational prompt significantly increased the stair climbing rate at the intervention site by 6.7% during the 6th-month intervention phase. Still, it did not affect the stair use in the 3rd month and the stair descending rate during the whole study period. In contrast, the stair rate at the control site remained similar to the baseline.

The 6.7% increase in stair climbing in our study was higher than a previous systematic review reported, which showed a median increase of 0.8% with a motivational sign only (15). Our results (6.7% increase in stair climbing) almost reached the increase in stair climbing in intervention involving a combination of motivational and directional signs, which were reported to increase stair climbing by 8.1% within a worksite setting (15). Our study is also in line with a previous study that reported that the changes in stair climbing could only be observed in the second phase (25). However, the decrease in stair climbing proportion in the first phase of our study might be caused by the decrease in total visitors during this period since people were more likely to choose the elevator in the less busy period (27–29). In comparison, previous studies required additional interventions in the second phase, such as improving the aesthetic of the stair or installing music (18, 30, 31). Our study’s increase in stair climbing could be observed without additional intervention. Therefore, we could identify that the positive influence on the stair climbing rate resulted from the repetition of prompts, which might be necessary to create sustainable habits in choosing the stair rather than the elevators. The stronger influence of motivational signs in our study could be attributed to the specific type of message, poster size, and the effect of co-production in designing the specific messages in the motivational sign (20, 32–34). Therefore, co-designing the intervention by involving the occupants of the buildings in designing large-size posters with specific messages since the early phase of intervention development could be suggested to improve the effectiveness as an alternative to using multiple intervention components.

Our study also found that employees easily adopted portable stair posters. This sign might solve overcoming adoption barriers reported by a previous study (25). Portable signage could still be located in the visible section of the building without interfering with the needs of corporate communications (25). Therefore, the placement of the stair posters could also be overtime during the study period.

Our study had several strengths. It employed a controlled design with two identical buildings and objective monitoring with less burden of observers, less observer bias, and less contamination bias. In addition, we also controlled total visitors as a confounder (20, 30, 32, 35, 36). However, there were several limitations of this study. Using double speed and reduced video recording resolution, our observers could not identify visitors’ characteristics, including gender, age, and whether visitors were students or employees. Previous studies found that gender, age, and status of visitors (students or employees) could influence the signage effect (29, 31, 36, 37). Therefore, future studies should consider controlling these confoundings. The difference in the number of floors, lift capacity, and the stair landing between the two buildings might also influence the visitors’ decision in choosing the stair for their journey, which could be examined in future studies. Whilst the number of flights was associated with health outcomes (10), the nature of the CCTV monitoring also did not allow for monitoring the number of flights the visitor took. We also examined the implementation process based on informal interviews and opinions, which could produce more naturalistic data but could be prone to bias and unreliable data (38). More rigorous mixed-method studies with a more representative sample involved in the development process of the intervention should also be considered in future process and impact evaluation studies. In addition, the effects of the intervention on individual or population health outcomes were also important to be examined in future studies.

## 5. Conclusion

Our study strengthens previous works on the usefulness of low-cost stair-use interventions at worksites using signage. Based on process evaluation using the RE-AIM framework, co-producing the intervention with employees was associated with the intervention’s effectiveness, adoption, and implementation. Effects of intervention on stair use amongst various genders, ages, and statuses of visitors, as well as effects on health outcomes, need to be elucidated in future studies.

## Data availability statement

The raw data supporting the conclusions of this article will be made available by the authors, without undue reservation.

## Ethics statement

The studies involving human participants were reviewed and approved by Medical and Health Research Ethics Committee Faculty of Medicine, Public Health, and Nursing Universitas Gadjah Mada. Written informed consent for participation was not required for this study in accordance with the national legislation and the institutional requirements.

## Author contributions

ZM: conceptualization, funding acquisition, and supervision. WW: conceptualization, funding acquisition, methodology, and writing–review and editing. MS: methodology, investigation, project administration, funding acquisition, and writing–review and editing. RW: conceptualization, methodology, investigation, formal analysis, writing–original draft, and writing–review and editing. DA: conceptualization, writing–review and editing, and supervision. All authors contributed to the article and approved the submitted version.

## Funding

This work was supported by the Community Empowerment Grant, Faculty of Medicine, Public Health, and Nursing, Universitas Gadjah Mada. This funding source had no role in the design of this study and will not have any role during its execution, analyses, interpretation of the data, or decision to submit results.

## Conflict of interest

The authors declare that the research was conducted in the absence of any commercial or financial relationships that could be construed as a potential conflict of interest.

## Publisher’s note

All claims expressed in this article are solely those of the authors and do not necessarily represent those of their affiliated organizations, or those of the publisher, the editors and the reviewers. Any product that may be evaluated in this article, or claim that may be made by its manufacturer, is not guaranteed or endorsed by the publisher.

## References

[1] Prince SA Elliott CG Scott K Visintini S Reed JL. Device-measured physical activity, sedentary behaviour and cardiometabolic health and fitness across occupational groups: a systematic review and meta-analysis. Int J Behav Nutr Phys Act. (2019) 16:30. doi: 10.1186/s12966-019-0790-9, PMID: 30940176 PMC6444868

[2] Cooper K Barton GC. An exploration of physical activity and wellbeing in university employees. Perspect Public Health. (2016) 136:152–60. doi: 10.1177/1757913915593103 26194136

[3] Haase A Steptoe A Sallis JF Wardle J. Leisure-time physical activity in university students from 23 countries: associations with health beliefs, risk awareness, and national economic development. Prev Med. (2004) 39:182–90. doi: 10.1016/j.ypmed.2004.01.028, PMID: 15208001

[4] Irwin JD. Prevalence of university students’ sufficient physical activity: a systematic review. Percept Mot Skills. (2004) 98:927–43. doi: 10.2466/pms.98.3.927-943, PMID: 15209309

[5] Meyer P Kayser B Kossovsky MP Sigaud P Carballo D Keller PF. Stairs instead of elevators at workplace: cardioprotective effects of a pragmatic intervention. Eur J Cardiovasc Prev Rehabil. (2010) 17:569–75. doi: 10.1097/HJR.0b013e328338a4dd, PMID: 20299999

[6] Arafa A Kokubo Y Shimamoto K Kashima R Watanabe E Sakai Y. Stair climbing and incident atrial fibrillation: a prospective cohort study. Environ Health Prev Med. (2022) 27:10. doi: 10.1265/ehpm.21-00021, PMID: 35288490 PMC9093618

[7] Chow BC Li S Zhu X Jiao J Quach B Baker JS. Effects of descending or ascending stair exercise on body composition, insulin sensitivity, and inflammatory markers in young Chinese women with obesity: a randomised controlled trial. J Sports Sci. (2021) 39:496–502. doi: 10.1080/02640414.2020.1829362 33012244

[8] Michael E White MJ Eves FF. Home-based stair climbing as an intervention for disease risk in adult females; a controlled study. Int J Environ Res Public Health. (2021) 18:603. doi: 10.3390/ijerph18020603, PMID: 33445686 PMC7828146

[9] Dunford EC Valentino SE Dubberley J Oikawa SY McGlory C Lonn E. Brief vigorous stair climbing effectively improves cardiorespiratory fitness in patients with coronary artery disease: a randomised trial. Front Sports Act Living. (2021) 3:630912. doi: 10.3389/fspor.2021.630912, PMID: 33665614 PMC7921461

[10] Sanchez-Lastra MA Ding D Dalene KE Del Pozo Cruz B Ekelund U Tarp J. Stair climbing and mortality: a prospective cohort study from the UK biobank. J Cachexia Sarcopenia Muscle. (2021) 12:298–307. doi: 10.1002/jcsm.12679, PMID: 33543604 PMC8061405

[11] Lee IM Paffenbarger RS Jr. Physical activity and stroke incidence: the Harvard alumni health study. Stroke. (1998) 29:2049–54. doi: 10.1161/01.str.29.10.2049 9756580

[12] Honda H Igaki M Hatanaka Y Komatsu M Tanaka S Miki T. Stair climbing/descending exercise for a short time decreases blood glucose levels after a meal in people with type 2 diabetes. BMJ Open Diabetes Res Care. (2016) 4:e000232. doi: 10.1136/bmjdrc-2016-000232, PMID: 27547414 PMC4964213

[13] Stenling A Moylan A Fulton E Machado L. Effects of a brief stair-climbing intervention on cognitive performance and mood states in healthy young adults. Front Psychol. (2019) 10:2300. doi: 10.3389/fpsyg.2019.02300, PMID: 31681096 PMC6803754

[14] Jennings CA Yun L Loitz CC Lee EY Mummery WK. A systematic review of interventions to increase stair use. Am J Prev Med. (2017) 52:106–14. doi: 10.1016/j.amepre.2016.08.014 27720340

[15] Bellicha A Kieusseian A Fontvieille AM Tataranni A Charreire H Oppert JM. Stair-use interventions in worksites and public settings – a systematic review of effectiveness and external validity. Prev Med. (2015) 70:3–13. doi: 10.1016/j.ypmed.2014.11.001, PMID: 25449692

[16] Bauman A Milton K Kariuki M Fedel K Lewicka M. Is there sufficient evidence regarding signage-based stair use interventions? A sequential meta-analysis. BMJ Open. (2017) 7:e012459. doi: 10.1136/bmjopen-2016-012459, PMID: 29183924 PMC5719260

[17] Adams J White M. A systematic approach to the development and evaluation of an intervention promoting stair use. Health Educ J. (2002) 61:272–86. doi: 10.1177/001789690206100308

[18] Olander EK Eves FF. Effectiveness and cost of two stair-climbing interventions-less is more. Am J Health Promot. (2011) 25:231–6. doi: 10.4278/ajhp.090325-QUAN-119, PMID: 21361807

[19] Grimstvedt ME Kerr J Oswalt SB Fogt DL Vargas-Tonsing TM Yin Z. Using signage to promote stair use on a university campus in hidden and visible stairwells. J Phys Act Health. (2010) 7:232–8. doi: 10.1123/jpah.7.2.232, PMID: 20484762

[20] Eckhardt MR Kerr J Taylor WC. Point-of-decision signs and stair use in a university worksite setting: general versus specific messages. Am J Health Promot. (2015) 29:291–3. doi: 10.4278/ajhp.120816-ARB-398, PMID: 24670074

[21] Glasgow RE Vogt TM Boles SM. Evaluating the public health impact of health promotion interventions: the RE-AIM framework. Am J Public Health. (1999) 89:1322–7. doi: 10.2105/AJPH.89.9.1322, PMID: 10474547 PMC1508772

[22] Zwarenstein M Treweek S Gagnier JJ Altman DG Tunis S Haynes B. Improving the reporting of pragmatic trials: an extension of the CONSORT statement. BMJ (Clin Res ed). (2008) 337:a2390. doi: 10.1136/bmj.a2390, PMID: 19001484 PMC3266844

[23] Eldridge SM Chan CL Campbell MJ Bond CM Hopewell S Thabane L. CONSORT 2010 statement: extension to randomised pilot and feasibility trials. BMJ (Clin Res ed.). (2016) 355:i5239. doi: 10.1136/bmj.i5239, PMID: 27777223 PMC5076380

[24] Seltman HJ. Experimental design and analysis. 1st ed. Pittsburgh, PA: Carnegie Mellon University (2012) Available at: https://www.stat.cmu.edu/~hseltman/309/Book/Book.pdf

[25] Bellicha A Kieusseian A Fontvieille AM Tataranni A Copin N Charreire H. A multistage controlled intervention to increase stair climbing at work: effectiveness and process evaluation. Int J Behav Nutr Phys Act. (2016) 13:47. doi: 10.1186/s12966-016-0371-0, PMID: 27067670 PMC4827230

[26] Vargas C Whelan J Brimblecombe J Allender S. Co-creation, co-design, co-production for public health – a perspective on definition and distinctions. Public Health Res Pract. (2022) 32:3222211. doi: 10.17061/phrp3222211, PMID: 35702744

[27] Kerr J Eves F Carroll D. Six-month observational study of prompted stair climbing. Prev Med. (2001) 33:422–7. doi: 10.1006/pmed.2001.0908, PMID: 11676583

[28] Webb OJ Eves FF Kerr J. A statistical summary of mall-based stair-climbing interventions. J Phys Act Health. (2011) 8:558–65. doi: 10.1123/jpah.8.4.558, PMID: 21597129

[29] Lewis AL Eves FF. Prompts to increase stair climbing in stations: the effect of message complexity. J Phys Act Health. (2012) 9:954–61. doi: 10.1123/jpah.9.7.954, PMID: 22971886

[30] Lewis A Eves F. Prompt before the choice is made: effects of a stair-climbing intervention in university buildings. Br J Health Psychol. (2012) 17:631–43. doi: 10.1111/j.2044-8287.2011.02060.x, PMID: 22248016

[31] Boutelle KN Jeffery RW Murray DM Schmitz MK. Using signs, artwork, and music to promote stair use in a public building. Am J Public Health. (2001) 91:2004–6. doi: 10.2105/ajph.91.12.2004, PMID: 11726383 PMC1446922

[32] Kerr J Eves FF Carroll D. The influence of poster prompts on stair use: the effects of setting, poster size and content. Br J Health Psychol. (2001) 6:397–405. doi: 10.1348/135910701169296, PMID: 12614513

[33] Kerr NA Yore MM Ham SA Dietz WH. Increasing stair use in a worksite through environmental changes. Am J Health Promot. (2004) 18:312–5. doi: 10.4278/0890-1171-18.4.312, PMID: 15011931

[34] Webb OJ Eves FF. Promoting stair climbing: effects of message specificity and validation. Health Educ Res. (2007) 22:49–57. doi: 10.1093/her/cyl045, PMID: 16763074

[35] Engelen L Gale J Chau JY Bauman A. Are motivational signs to increase stair use a thing of the past? A multi-building study. Health Promot J Austr. (2017) 28:178–84. doi: 10.1071/HE16107, PMID: 28264762

[36] Yoon A Choi S Mun J Hong J Hahn D Kang M. Motivational signage increases stair usage on a Hispanic serving institution. J Am Coll Health. (2020) 68:236–41. doi: 10.1080/07448481.2018.1539000, PMID: 30570444

[37] Nomura T Yoshimoto Y Akezaki Y Sato A. Changing behavioral patterns to promote physical activity with motivational signs. Environ Health Prev Med. (2009) 14:20–5. doi: 10.1007/s12199-008-0053-x, PMID: 19568864 PMC2684766

[38] Swain J King B. Using informal conversations in qualitative research. Int J Qual Methods. (2022) 21:160940692210850. doi: 10.1177/16094069221085056

